# Diagnosis of localized cutaneous leishmaniasis and treatment outcomes following carbon dioxide cryotherapy performed by health centre staff: A pilot implementation study in Ethiopia

**DOI:** 10.1371/journal.pgph.0006930

**Published:** 2026-09-09

**Authors:** Feleke Tilahun Zewdu, Kassahun Alemu, Saba Lambert, Bisrat Misganaw, Zemed Biyazn Abebe, Eyob Fekadu Shegaw, Tedros Nigusse, Galana Mamo Ayana, Eshetu Molla, Rachel L. Pullan, Jennifer Palmer, Catherine Pitt, Yohannes Hailemichael, Michael Marks, Endalamaw Gadisa, Stephen L. Walker

**Affiliations:** 1 Department of Epidemiology and Biostatistics, Institute of Public Health, College of Medicine and Health Sciences, University of Gondar, Gondar, Ethiopia; 2 Boru Meda General Hospital, Amhara, Ethiopia; 3 Division of Malaria and Neglected Tropical Diseases Research, Armauer Hansen Research Institute, Addis Ababa, Ethiopia; 4 Faculty of Infectious and Tropical Diseases, London School of Hygiene & Tropical Medicine, London, United Kingdom; 5 ALERT Comprehensive Specialized Hospital, Addis Ababa, Ethiopia; 6 Faculty of Public Health and Policy, London School of Hygiene & Tropical Medicine, London, United Kingdom; 7 Hospital for Tropical Diseases, University College London Hospital, London, United Kingdom; 8 Division of Infection and Immunity, University College London, London, United Kingdom; University of Colorado Anschutz Medical Campus: University of Colorado - Anschutz Medical Campus, UNITED STATES OF AMERICA

## Abstract

In Ethiopia, the incidence of cutaneous leishmaniasis (CL) is estimated to range from 20, 000–30, 000 new cases due to *Leishmania aethiopica*. Children and young adults, in rural communities with limited access to health care are most affected. The current standard of care, pentavalent sodium stibogluconate is only administered in specialist centres, has limited effectiveness and is associated with severe adverse effects. Cryotherapy is recommended in national guidelines at the primary care level to treat uncomplicated CL, but is rarely available due to limited numbers of trained clinicians and lack of resources. We co-developed and evaluated a decentralised intervention, administered at a rural health centre, using carbon dioxide (CO_2_) cryotherapy, which is available for the treatment of cervical dysplasia. A pragmatic clinical trial design was used, providing training at the health centre that combined theoretical instruction with direct patient assessment. Study clinicians recruited patients with suspected CL who had a slit-skin smear performed with confirmation by expert microscopists. The diagnostic accuracy of trained clinical and laboratory staff at a rural health centre were compared to experts. Individuals aged ≥12 years with parasitologically confirmed uncomplicated CL received CO_2_ cryotherapy. Participants were followed until Day 90. Cure was defined as complete re-epithelialization of the index lesion. The study was registered in the Pan African Clinical Trials Registry with trial number PACTR202605616785682. https://pactr.samrc.ac.za/TrialDisplay.aspx?TrialID=41111. We trained seven clinicians and laboratory professionals at Keteteya Health Centre. Of 331 individuals who attended the clinic with skin disease, 158 (47.7%) were clinically diagnosed by trained clinical staff with CL. Overall 123 (78%) had laboratory confirmed CL and of these 85 (53.8%) received CO_2_ cryotherapy. By Day 90, 68 of 77 (88.3% (95% CI: 78.5–94.1%)) were cured. Adverse events were mild and transient, with no individual discontinuing treatment. Trained health centre clinical and laboratory staff were able to accurately diagnose CL. CO_2_ cryotherapy was safe and efficacious when administered by trained staff. A training programme to facilitate CO_2_ cryotherapy appears to be a feasible approach to decentralizing CL diagnosis and treatment to primary healthcare facilities in endemic areas of Ethiopia.

## Author summary

Cutaneous leishmaniasis (CL) is a neglected tropical disease (NTD) of the skin caused by species of the genus *Leishmania*. It is endemic in Ethiopia’s highland regions. Affected individuals often face significant barriers to accessing care due to the limited numbers of trained health professionals and long distances to diagnostic and treatment centres. Treatments for CL, including sodium stibogluconate and liquid nitrogen cryotherapy, are challenging to implement in rural settings. Carbon dioxide cryotherapy is now widely available for cervical dysplasia in primary health centres in Ethiopia and could potentially be repurposed for the management of CL. Our study evaluated the efficacy of carbon dioxide cryotherapy for the treatment of CL when administered by trained health centre staff. Eighty-five individuals with confirmed CL were treated with carbon dioxide cryotherapy and 68 (88.3%) were healed at Day 90. These findings suggest that carbon dioxide cryotherapy is efficacious when administered by trained health centre staff. Controlled trials are warranted to confirm these findings and to inform implementation strategies.

**Evidence Box**

**Evidence before this study**

We searched PubMed without restrictions on language or publication year, using search terms including “cutaneous leishmaniasis,” and “cryotherapy”. We identified 20 studies evaluating liquid nitrogen-based cryotherapy for the treatment of cutaneous leishmaniasis. Eighteen assessed treatment effectiveness in a hospital setting, one in a health post and one in a health centre. Only two studies assessed carbon dioxide-based cryotherapy for cutaneous leishmaniasis, both at the hospital level.

These studies generally reported that cryotherapy is a safe and effective treatment for cutaneous leishmaniasis, with cure rates between 25–100%, depending on species, lesion characteristics, and treatment protocols. No study evaluated the use of carbon dioxide cryotherapy at the health centre level, either in isolation or as part of a decentralized care pathway.

**Added value of this study**

In this prospective study we report outcomes for patients diagnosed and managed with CO2 cryotherapy at primary health facilities in Ethiopia. We systematically assessed diagnostic accuracy using standardized metrics. We found that the accuracy of both initial clinical assessment and laboratory confirmation was high in this setting with sensitivities and specificities above 90%. Overall, 79.11% of patients with confirmed cutaneous leishmaniasis were treated at the health facility. Treatment outcomes were favorable with 88.3% of patients cured by day 90 and 76.9% experiencing mild and transient treatment associated adverse events. No participants withdrew due to adverse events. These findings support task-shifting and the integration of carbon dioxide cryotherapy into primary health-care services as a viable treatment strategy for cutaneous leishmaniasis.

**Implications of all the available evidence**

Cryotherapy appears to be a viable treatment strategy for uncomplicated cutaneous leishmaniasis. The available evidence supports the potential feasibility of decentralized diagnosis and treatment of uncomplicated cutaneous leishmaniasis to primary health-care facilities. Expanding access to diagnosis and lesion directed treatments should reduce delays in care, increase treatment coverage, and lower both patient and health system costs. Further research is needed to guide the optimal scale-up of this approach.

## Introduction

Cutaneous leishmaniasis (CL) is a neglected tropical disease (NTD) of the skin which poses substantial public health challenges in Ethiopia. Approximately 29 million Ethiopians live in settings endemic for CL, and 20,000–30,000 new cases, primarily caused by *Leishmania aethiopica* [1,2], are estimated to occur each year [1]. Rural communities with limited healthcare access bear the highest burden of CL [3,4]. CL is associated with reduced health-related quality of life (HRQoL) [5] and high – sometimes catastrophic – costs of care-seeking, in part because care is only available at a small number of specialized treatment centres, which are distant from many affected individuals’ homes [6].

The choice of treatment depends on lesion characteristics (size, number and location), the species of *Leishmania*, the clinical phenotype, risk of mucosal involvement, patient preference, and treatment availability. CL caused by *L. aethiopica* has three clinical phenotypes: localized CL (LCL), mucocutaneous leishmaniasis (MCL), and diffuse CL (DCL). Severe LCL, MCL and DCL require systemic therapy only available at referral centres in Ethiopia. Sodium stibogluconate (SSG) is recommended and usually administered on an inpatient basis [6]. Parenteral SSG administration may be associated with severe adverse effects [7].

Lesion-directed therapy (LDT) is recommended for uncomplicated LCL [8,9]. LDT does not require inpatient monitoring but is not currently available in primary healthcare facilities in Ethiopia. Liquid nitrogen cryotherapy is the main LDT recommended in Ethiopia and elsewhere [7,10] but is often only available in specialized CL treatment centres because procurement and safe storage poses logistical challenges, and administration requires significant training [11].

An alternative cryogen, CO_2_, has been used to treat a range of skin conditions and has recently become widely available at health centres in Ethiopia to treat cervical dysplasia. Studies in Yemen [12] and Egypt [13] reported that CO_2_ cryotherapy administered by dermatologists could effectively treat CL. We recently confirmed this finding in Ethiopia at Boru Meda General Hospital, where 14 of 17 (87.5%) individuals with LCL (children and adults) treated with CO_2_ cryotherapy by a dermatologist were cured by Day 90 [14].

Leveraging the existing availability and use of CO_2_ cryotherapy within health centres to treat uncomplicated LCL offers the potential to greatly increase access to care for CL; however, the ability of health centre staff to treat CL with CO_2_ cryotherapy has not previously been evaluated. We therefore conducted a prospective evaluation of a comprehensive health system intervention to support the diagnosis and treatment of CL using CO_2_ cryotherapy in health centres in Ethiopia.

## Methods

To improve access to CL diagnosis and treatment, we co-developed an intervention that incorporated CL diagnosis and treatment of eligible patients using CO_2_ cryotherapy delivered within the primary healthcare system. The diagnostic and treatment components were developed by dermatovenereologists, infectious disease specialists, microbiologists, and parasitologists.

### Open science

This trial was registered in the Pan African Clinical Trials Registry (PACTR202605616785682) after data collection and analysis. Because the study used a pragmatic/implementation design, we initially believed registration was unnecessary. After submitting to PLOS Global Public Health, the journal required registration, so we registered the trial post-data collection and added the registration number. The authors confirm that all ongoing and related trials for this intervention are registered.

### Study setting

Data collection and case recruitment were conducted by clinicians at KHC between June 24, 2024 and January 01, 2025. Ketetya Health Centre (KHC), Kalu District in Amhara Regional State, serves a population of approximately 47,000. It has 16 clinical staff who provide emergency and non-urgent care. Services include maternity care, outpatient clinics, and cervical cancer screening and treatment, including CO_2_ cryotherapy, which is easy to administer [15]. The laboratory services available include rapid diagnostic tests and microscopy for malaria, tuberculosis, and leprosy. Kalu District is endemic for CL. The closest specialized dermatology service and CL treatment centre is at Boru Meda General Hospital, 76 km away.

### Community engagement

Two stakeholder feedback meetings were conducted: the first meeting was a sensitization meeting held before the start of the co-developed intervention, that included community leaders, health extension workers, religious leaders, health centre heads, district health bureau directors, previously treated CL cases, Kebele association leaders, individuals with skin disease lived experience and members of the leprosy affected person’s association as well as members of the research teams in the Keteteya cluster. The meeting served to introduce the study and reiterate the findings of the formative work [10] that informed the design of the intervention.

The second meeting was with Keteteya health extension workers, the head of the Kalu District Health Office, and NTD officers, and the head of the Zonal health office and officers. The policy level engagement included regional and national program leaders, along with delegates from implementing partners and civil society organizations.

### Training of KHC Staff

Clinical and laboratory staff were trained using resources developed by the research team on skin disease and CL, alongside previously developed materials [10,14]. Training combined theoretical sessions with supervised practical exercises with individuals affected by CL and in the use of slit-skin smear. Clinical vignettes were used to reinforce key learning objectives. Participating KHC staff comprised three laboratory technicians, two nurses, and two health officers. The four KHC clinical staff were trained in the application of CO_2_ cryotherapy for CL; two were already trained in performing cryotherapy for cervical dysplasia at the health centre. The three laboratory professionals had training on slit-skin smear.

#### Clinician training.

Clinical training was delivered by a dermatology expert based at Boru Meda General Hospital (FTZ), two physicians (DY and HH), and two laboratory professionals (EFS and ZBA). Clinical training covered the initial assessment and treatment of common skin disorders, recognition of CL, and the appropriate use of CO_2_ cryotherapy, as well as relevant study-specific elements, including informed consent and data collection. Following training at KHC, each KHC clinician had one day of practical training at Boru Meda General Hospital, where they assessed 21 patients with parasitologically-confirmed CL and administered CO_2_ cryotherapy to four patients under the supervision of one of the trainers. CO_2_ cryotherapy administration is described in detail below.

A further 10 days of practical training at KHC, supervised by the study team, were then undertaken. This included supervised clinical assessment of individuals with skin disorders and practicing study procedures. KHC clinicians were supervised in the assessment of twenty-two individuals with suspected CL who had a slit-skin smear performed. Of these, 12 individuals were treated with CO_2_ cryotherapy by KHC clinicians supervised by one of the trainers. Feedback was provided by the trainers to the KHC clinicians throughout the three stages of training to improve knowledge and skills. A final evaluation was conducted using a standard checklist covering a range of key areas, including their ability to independently apply CO_2_ cryotherapy (S1 File).

#### Laboratory personnel training.

Laboratory training was provided by two experienced laboratory-technicians who each had > 2 years of research experience at the AHRI *Leishmania* research laboratory and routinely processed >400 slit-skin smears annually. Slit-skin smear microscopy was implemented at KHC as a point of care diagnostic technique for the confirmation of CL. In brief, a 3–5 mm incision was made at the edge of an active lesion to a depth of 2–3 mm using a size 15 scalpel blade. Tissue fluid was smeared onto a glass slide, air-dried, fixed with methanol, and stained with Giemsa. Slides were subsequently examined under a light microscope to visualize amastigotes.

Training was delivered through two days of practical laboratory sessions at Boru Meda General Hospital. Trainees observed slit-skin smear staining, microscopy and preparation of a report. Each trainee was observed by colleagues and trainers while performing the procedures. Following this, trainees received a further 10 days of supervised practical training at KHC. During this period, laboratory trainees performed slit-skin smears on 22 individuals with suspected at KHC. Experts provided continuous feedback throughout the training to help improve the trainees’ skills. At the end of the training, the laboratory trainees were evaluated by experts using a standard checklist to assess their ability to perform and process slit-skin smears (S2 File).

### Participant recruitment

Individuals with a skin problem who presented to KHC were evaluated by one of the four trained KHC clinicians. All individuals with suspected CL were invited to participate in the study. Individuals diagnosed with skin conditions other than CL were treated free of charge with appropriate medication supplied by the study team. Individuals with conditions deemed beyond the scope of KHC, including those with complex CL and other study exclusion criteria, were referred to Boru Meda General Hospital.

For individuals with suspected CL, a slit-skin smear was performed, stained with Giemsa, and 100 fields examined at 100x oil immersion objective (Olympus CX23, Model Number CX23LEDRFS1). All slit-skin smear slides were stored for independent assessment each week by the two laboratory experts who were blinded to the KHC results. At the end of the study, *Leishmania* DNA polymerase chain reaction (PCR), followed by restriction fragment length polymorphism (RFLP) for species confirmation, was performed on slit-skin smear samples (S3 File).

#### Inclusion criteria for CO_2_ cryotherapy.

In the absence of a formal guideline, we developed criteria for individuals who would be eligible for CO_2_ cryotherapy at KHC. For this study, we considered CO_2_ cryotherapy suitable for individuals 12 years and above with four or fewer CL lesions, each of which was less than 5 cm in diameter and more than 2 cm from a mucosal surface [6,14]. Lesion diameter and distance from a mucosal surface was measured using a disposable tape measure (S4 File).

Individuals with parasitologically confirmed CL (i.e., *Leishmania* amastigotes visualised in slit-skin smears) who met the above criteria, were offered CO_2_ cryotherapy performed by the trained KHC clinicians. Individuals with CL who declined CO_2_ cryotherapy or did not meet the inclusion criteria were referred to Boru Meda General Hospital.

### CO_2_ Cryotherapy administration

CO_2_ cryotherapy was applied using the Medgyn Cryotherapy System MGC-200 as described previously [14,16]. The system uses compressed CO_2_ to cool a metallic tip to approximately –78°C. Three different tip sizes were available: 1 cm, 3 cm, and 5 cm in diameter. The following CO_2_ cryotherapy regimen was employed: an appropriate size of cryo-tip was cooled until ice appeared on the tip (for a maximum of 15 seconds, using a timer); the cooled tip was applied to the CL lesion for 40 seconds, followed by a thawing period of 20 seconds. The cryo-tip was cooled again and a second application of 40 seconds performed. Participants were assessed every two weeks by the study clinicians at KHC [14]. CO_2_ cryotherapy was repeated at each visit (up to a maximum of six visits) if further cryotherapy was deemed necessary by the dermatology expert (FTZ). Lesions not healed after six cryotherapy sessions were deemed unresponsive and participants were referred to Boru Meda General Hospital.

### Data collection

At enrolment, demographic and clinical data were collected by the KHC clinicians and a representative digital image of each participants’ skin problem was taken. At each follow-up visit, and on Day 90, both the participant and the research clinician provided an assessment of lesion healing using an ordinal scale (worse, no change, somewhat better, better, and healed), measured lesion size and documented any adverse events. The Amharic version of the Dermatology Life Quality Index (DLQI) [17] was administered to participants aged ≥16 years at enrolment and repeated at Day 90. The Patient and Observer Scar Assessment Scale v 2.0 (POSAS 2.0) [18] was completed with all participants who were deemed cured (defined below) at Day 90. The research team attempted to contact participants who did not attend for planned visits. Participants who were not assessed at Day 90 were classified as lost to follow-up.

### Outcome assessment

The primary outcome was cure at Day 90 following the first application of CO_2_ cryotherapy. We defined cure using a global assessment by the dermatologist of complete re-epithelialization for ulcerated lesions, complete flattening of non-ulcerated lesions, and reductions in induration and redness [19]. Non-ulcerated macular lesions were assessed for resolution of erythema. For participants with more than one CL lesion at enrollment, the largest lesion was selected for assessment. Clinical signs such as pigmentary change at Day 90 were recorded as hypopigmentation or hyperpigmentation. Participants were also asked about symptom relief, changes in appearance, and overall treatment satisfaction [20].

Furthermore, to minimize assessment bias in the primary outcome, lesion healing was evaluated after completion of data collection and before statistical analysis through an independent, blinded, lesion-by-lesion assessment conducted by experienced dermatovenereologists using standardized clinical photographs [21].

Adverse effects associated with the application of CO_2_ cryotherapy were documented during the final follow-up visit of the treatment course. The study clinicians asked about skin changes including changes in skin pigmentation, the presence or absence of redness, swelling, and blistering. Secondary bacterial infection was diagnosed clinically without microbiological confirmation. The KHC clinical team encouraged participants to report any other symptoms experienced following cryotherapy.

### Data management and analysis

As this was a single-arm pilot study, we did not calculate a formal sample size. We aimed to deliver the intervention over a period of eight months, which we considered sufficient to assess the feasibility of the intervention.

We evaluated both clinical and laboratory diagnosis by comparison to an expert reference standard.

To assess the accuracy of the initial clinical examination, an expert, unaware of the KHC staff diagnosis, reviewed each individual with suspected CL. We calculated the proportion of individuals for whom the expert assessment agreed with the initial clinical classification.

To assess the performance of KHC laboratory staff slit-skin smear assessment, each slides was re-read by one of the laboratory trainers. We calculated the sensitivity, specificity, positive predictive value (PPV) and negative predictive value (NPV) of microscopy performed by the KHC laboratory staff compared to expert microscopists.

Descriptive statistics were used to summarize the demographic and clinical data of the study participants in the CO_2_ intervention. Proportions and 95% confidence intervals (IC) were estimated using a generalized linear mixed model (GLMM) with a binomial distribution and logit link function, accounting for repeated measurements with participant-level random effects. CI were calculated on the logit scale and back-transformed to the probability scale. Furthermore, we reported the proportion of participants who were cured by Day 90 alongside changes in DLQI scores from baseline to Day 90. Higher DLQI scores indicate worse HRQoL. To compare the median change in DLQI between Day 1 and Day 90 without assuming normality, we used the Wilcoxon signed-rank test statistics.

We collected data electronically into a GCP-compliant REDCap database and analyzed data using STATA version 17. Missing data or one who did not complete their follow ups were excluded from the analysis.

### Inclusivity in global research

Additional information regarding the ethical, cultural, and scientific considerations specific to inclusivity in global research is included in the Supporting Information (S4 File).

### Ethics statement

Ethical approval was obtained from the University of Gondar (Ref: CMHSSH-UOG IRERC/44/05/2024), the London School of Hygiene & Tropical Medicine (LSHTM Ethics Ref: 29017) and the AHRI/ALERT Ethics Review Committee (Ref: PO-26–20). Written informed consent was obtained from all adult participants and KHC staff. For individuals <18 years of age, consent was obtained from a responsible adult (parent or legal guardian) and assent was additionally sought from participants aged 12–17 years. All information and consent procedures were conducted in Amharic. The funder had no role in the design, conduct or analysis of the study.

## Results

### Clinical accuracy

**Fig 1:**
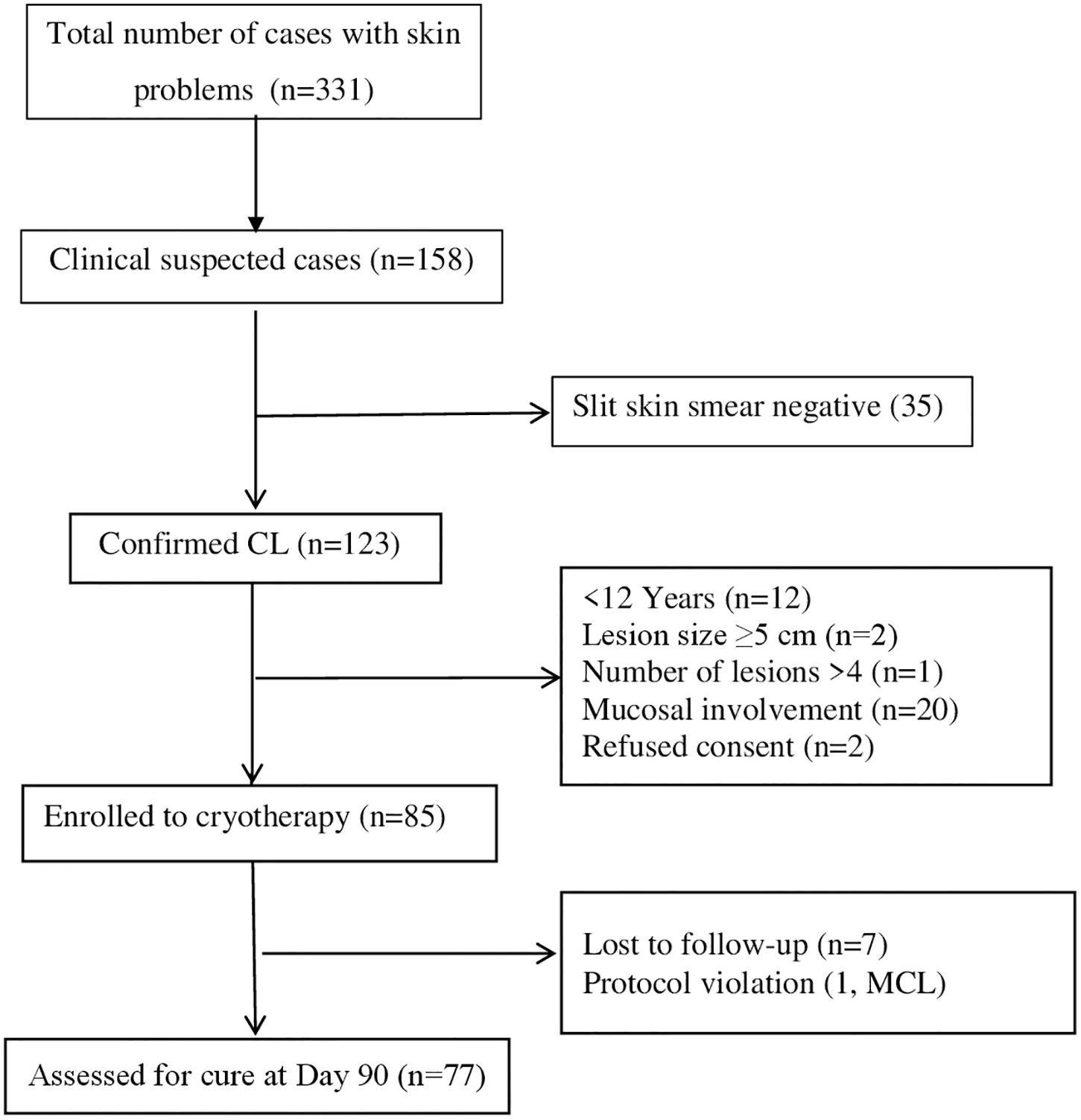
CONSORT diagram.

From June 2024 to January 2025 (inclusive), KHC clinicians assessed 331 individuals with a skin condition at KHC, of whom 158 (47.7%) were identified as possibly having CL (Fig 1, Table 1). Based on review of lesion photographs, 6 individuals (3.6%) whom the KHC clinicians diagnosed with possible CL were judged not to have the condition. No individual originally classified as having a skin condition other than CL was reclassified as having CL on review. Cases misdiagnosed by KHC clinicians as CL were pyogenic skin abscess (n = 2), and one case each of lepromatous leprosy, lichenified discoid eczema, morphea and nodulocystic acne. The sensitivity of the initial clinical evaluation for clinically suspected CL was 100% and specificity was 96.4%, with positive and negative predictive values of 96.2% and 100%, respectively.

**Table 1:** The diagnostic accuracy of KHC clinicians and laboratory testing compared to expert review by clinicians and laboratory experts.

		Expert review		
**Clinical**		**CL**	**Not CL**	**Total**
**KHC clinicians**	**CL**	152	6	158 (47.7%)
	**Not CL**	0	173	173 (52.3%)
	**Total**	152	179	331
**Laboratory**		**Slit-skin smear positive**	**Slit-skin smear negative**	**Total**
**KHC lab**	**CL**	123	6	129 (73.3%)
	**Not CL**	1	46	47 (26.7%)
	**Total**	124 (79.6%)	52 (20.5%)	176

### Laboratory accuracy

Slit-skin smear microscopy was performed on 158 individuals. Of these, the KHC laboratory staff reported 113 (71.5%) as positive and 45 (28.5%) as negative, whilst the expert laboratory technicians reported 123 (77.9%) as positive and 35 (22.1%) as negative. The sensitivity of slit skin smear performed by KHC laboratory staff was 95.3% and specificity 97.9%, with a positive predictive value of 94.9% and a negative predictive value of 97.5% (Table 1). Of the 123 positive slit-skin smears reported by the expert laboratory technicians, 118 were tested by PCR of which 86 (72.9%) had *Leishmania* DNA detected, all of which were confirmed as *Leishmania aethiopica* DNA.

### Carbon dioxide cryotherapy eligibility

Of the 123 individuals with CL confirmed on slit-skin smear, 35 (28.5%) were judged to be ineligible for CO_2_ cryotherapy. This may be due to the clinical characteristics of the lesions, including MCL, lesions located within 2 cm of the mucosal margin, the presence of more than four lesions, and lesion sizes greater than or equal to 5 cm. In addition, one participant with MCL was initially misclassified as having LCL, and two eligible participants declined to participate in the treatment trial.. Ultimately, 85 individuals with 116 skin lesions were enrolled and received CO_2_ cryotherapy (Fig 1). Of these, 44 (51.2%) were female, 40 (47.1%) were children (12–18 years old), and the median age was 23 years (IQR 12–32). Fifty (58.1%) had used traditional treatment before enrolment in the study and the median duration of the index lesion was 12 months (IQR 5–12) (Table 2).

**Table 2:** Demographic and clinical characteristics for participants enrolled to receive carbon dioxide-based cryotherapy.

Characteristic	N (%)
**Sex**	
Male	42 (48.8)
**Age**	
Years (median, IQR)	23 (12, 32)
**Previous history of treatment**	
Any	50 (58.1)
Traditional treatment	47 (54.65)
Medical treatment*	3 (3.5)
**HIV antibody status**	
Positive	3 (3.5)
Negative	82 (96.5)
**Number of lesions**	
1	68 (80.2)
2	9 (10.5)
3	3 (3.5)
4	5 (5.8)
**Site of the index lesion**	
Cheek	34 (40)
Nose	29 (29)
Forehead	8 (8.2)
Forearm	5 (5.9)
Chin	4 (4.7)
Arm	2 (2.4)
Elbow	2 (2.4)
Eyelid	1 (1.2)
Finger	1 (1.2)
**Size of the index lesion**	mm (median, IQR)
	20 (10, 27)
**Morphology of the index lesion**	
Patch	24 (27.9)
Papule	5 (5.8)
Nodule	6 (7.0)
Plaque	51 (59.3)
**Ulceration of index lesion**	
Yes	49 (57.0)
**Median duration of CL lesion (**months)	(median, IQR)
	12 (5, 12)

### Outcomes of carbon dioxide cryotherapy

**Fig 2:**
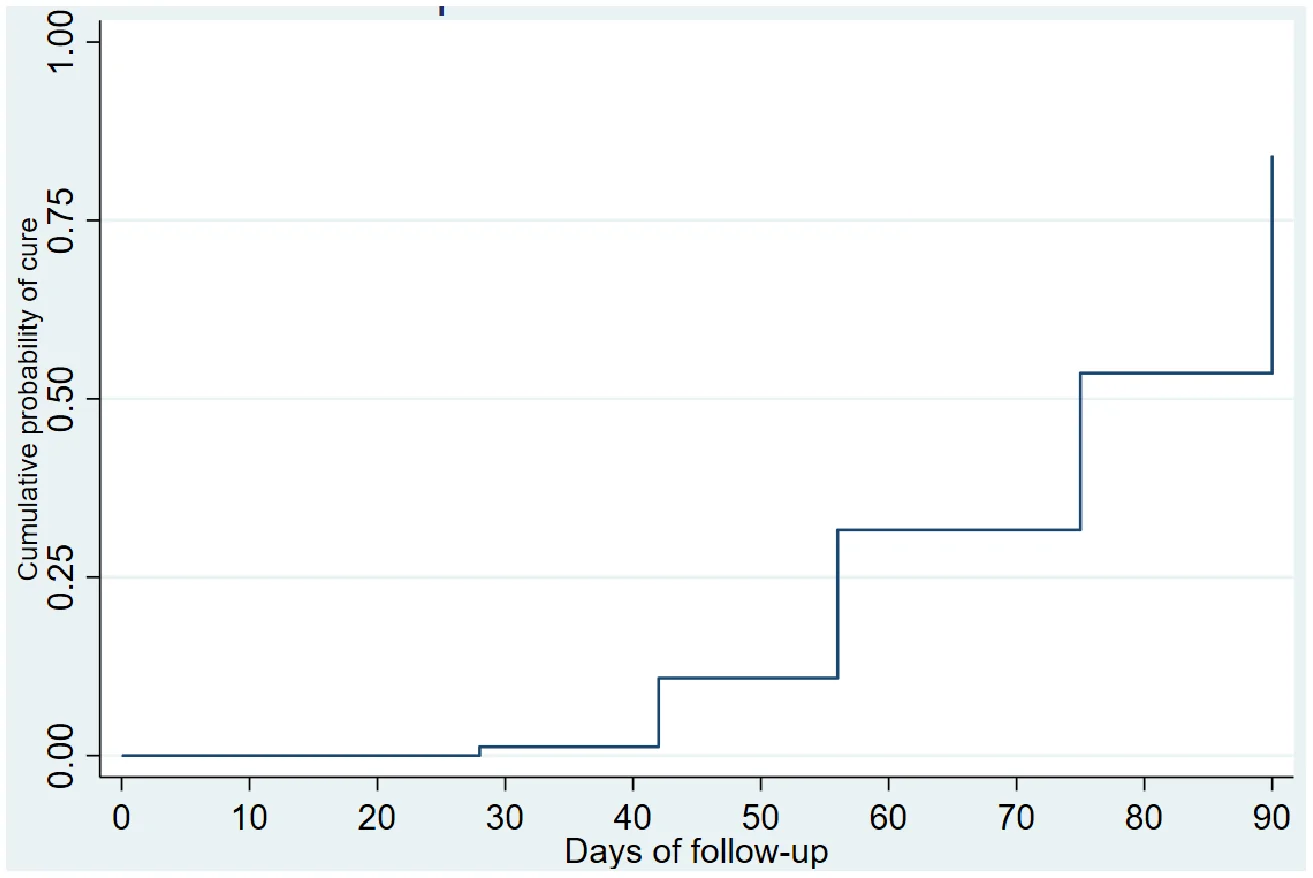
Time to cure in participants treated with CO2 cryotherapy.

Eight participants were lost to follow-up. Of the participants seen at day 90, 71 of 77 (92.2%) had received six sessions of CO_2_ cryotherapy. The dermatology expert (FTZ) assessed 68 of 77 (88.3%, 95% CI: 78.5-94.1%) as healed (Fig 2). Four (5.2%) participants were deemed better, and 5 (6.5%) somewhat better. No participants were classified as unchanged or worse. At day 90, no participants assessed their condition as healed. 73 (94.8%) participants reported their condition as better, and 4 (5.2%) as somewhat better.

### Health-related quality of life

Thirty-five participants had a DLQI performed at enrolment and Day 90, all of whom were assessed as cured. In individuals who were deemed cured by physicians at Day 90, the median DLQI score was 8 (IQR 5–13) at enrolment and 0 (IQR 0–1) at Day 90 (p < 0.001). At Day 90, the median participant-rated component of the POSAS score was 7 (IQR 6–8). The median observer (physician-rated) component was 6 (IQR 5–9).

### Adverse events

Amongst people seen at Day 90, the most frequent adverse events reported were swelling at the site of application (n = 59/77, 76.9%), pain at the treatment site (n = 58, 74.4%), erythema (n = 51/77, 66.7%), hyperpigmentation (n = 45/77, 59.0%), and hypopigmentation (n = 21/77, 26.9%). Blistering (n = 14/77, 17.95%), suspected secondary bacterial infection (n = 15, 19%), necrosis (n = 4, 5.1%), and swelling not on the application site (n = 3, 3.9%) occurred less frequently (Table 3). No participants withdrew due to adverse events.

**Table 3:** Adverse events associated with CO_2_ cryotherapy.

Adverse event	n (%)
Treatment site pain	58 (74.4%)
Pigmentary changes	
Normal skin colour	11 (14.1%)
Hyperpigmentation	45 (59%)
Hypopigmentation	51(66.7%)
Redness	59 (76.9%)
Localised swelling	14 (18%)
Blistering	4 (5.1%)
Necrosis	15 (19%)
Secondary bacterial infection	3 (3.9%)
Swelling not on the application site	21 (26.9%)
Other	4 (5.1%)

## Discussion

This study shows that high clinical and laboratory diagnostic accuracy can be achieved in routine care and that CO_2_cryotherapy is a safe, scalable, and effective treatment for CL, yielding high cure rates, substantial HRQoL improvement, and acceptable adverse events in a resource-limited setting. Health centre staff were able to correctly diagnose CL and identify patients for whom CO_2_ cryotherapy treatment was appropriate. Of those eligible, 88.3% were cured by Day 90 of follow-up. We leveraged the use of CO_2_ cryotherapy established for the national cervical cancer control program to implement our intervention.

We have demonstrated that diagnosis and management of CL can be decentralized to a health centre and that this model of care can provide high cure rates for individuals with uncomplicated LCL in Ethiopia. Using this broader infrastructure provides an opportunity to expand treatment for LCL in the region which is critical to overcome the substantial barriers to care experienced by many patients. Reports of a an accompanying analysis of participants, health workers, and health system managers perspectives on the pilot will be reported separately but in brief all groups expressed strong support for CO_2_ cryotherapy, viewing it as effective, acceptable, and financially beneficial, particularly in rural areas where distance and limited awareness hinder access to treatment. Managers noted that scale-up is feasible but requires policy commitment, adequate funding, and careful planning.

Delivering decentralized management of CL requires several processes to work efficiently. Health care workers must be able to identify potential cases from the large group of individuals presenting with skin disorders, an appropriate diagnostic sample must be taken and interpreted accurately, patients suitable for treatment must be identified and those requiring more complex care referred elsewhere, and treatment must be available and delivered to a high standard. Our data suggest each of these steps was largely achieved. Review by a dermatology expert identified no cases where possible CL was missed (among people presenting with a skin problem) by the health centre staff, whilst slit-skin smear microscopy performed by health centre staff demonstrated high diagnostic accuracy with a sensitivity of 95.3% and specificity of 97.9%. Research in rural Ethiopia indicates that slit-skin smear microscopy remains the standard diagnostic method for CL due to its simplicity, low cost, and feasibility, despite its moderate sensitivity compared with histopathology or molecular techniques, which are not available in health centers [2,22]. Two-thirds of participants with confirmed LCL were eligible for CO_2_ cryotherapy, and most who received it were cured. Individuals who were cured had a significant improvement in HRQoL. Collectively, these data suggest that early identification and treatment of CL is possible and may avoid the need for more complex systemic therapies.

The 88.3% (95%CI: 78.5-94.1%) cure rate observed in this study is similar to the 87.5% cure rate found in our smaller prospective study conducted at the specialised leishmaniasis treatment centre at Boru Meda General Hospital [14], suggesting care can be decentralised whilst maintaining similar outcomes. The overall cure rate was slightly lower than seen in Yemen, where all participants were lesion-free at Day 90 [12]. These differences may reflect differences in study populations and lesion characteristics, the frequency of administration and expertise of health providers administering cryotherapy, and differences associated with different *Leishmania* species. We observed a higher cure rate with CO_2_ cryotherapy than we had observed in hospital-based cohort study in which participants received the standard of care [12]. This is likely to be attributable to the smaller, less complex lesions affecting individuals in the current study. Overall, our results support CO_2_ cryotherapy as a safe and effective treatment for uncomplicated CL that can be delivered at primary healthcare facilities [23].

Our study shows that decentralizing CL management using CO_2_ cryotherapy is a highly effective strategy for bridging the treatment gap in rural, resource-limited settings. Targeted training of primary health centre clinicians and laboratory staff enabled a high clinical detection rate among patients with skin disease and an 88.3% cure rate by Day 90, with only mild, transient adverse events. These findings indicate that clinical efficacy in decentralized models depends on human capacity rather than type of facility, consistent with evidence that primary health centres can manage CL when supported by supervision and reliable supplies [24-26]. We propose a framework in which primary healthcare facilities provide CO_2_ cryotherapy for LCL, while specialist institutions oversee complex cases. This model could expand timely access, maintain oversight for complex cases, and strengthen CL control through a scalable, patient-centered approach; however, its success would require robust training, supervision, and supply systems [24,25].

The strengths of our study include routine dermatology professional expert supervision and direct provision of feedback to the clinicians. The provision of regular feedback on diagnostic performance from the laboratory experts and the waiving of CL-related payments. Prospective data collection embedded in a real-world primary healthcare setting in Ethiopia, and the use of standardised definitions for clinical descriptions, outcome measures, and patient-reported outcome measures are additional strengths.

There were also several limitations. A proportion of participants did not return for their Day 90 follow-up assessment. This may have been due to treatment failure, participants feeling cured before completing follow-up, limited transportation, or competing personal and economic responsibilities. This was an uncontrolled study, and therefore, spontaneous lesion healing may have influenced our estimate of the efficacy of the intervention. Despite these limitations, CO2 cryotherapy appeared promising with a high cure rate and a low rate of complications. This was the first attempt to decentralise cryotherapy, and we therefore pragmatically opted not to include children <12 years of age. Future studies in younger children would be valuable. Finally, the research team provided support during the study, including guiding some clinical decision-making. Larger prospective studies without dedicated support are required to understand the real-world effectiveness of CO_2_ cryotherapy.

In Ethiopia and many other CL endemic settings, access to treatment for CL urgently requires improvement [11,26]. Our findings suggest that decentralized diagnosis and management with CO_2_ cryotherapy can be successfully implemented in primary healthcare settings. The widespread scale-up of CO_2_ cryotherapy for cervical cancer in many settings provides an opportunity to transform access to CL treatment. Further studies are needed to understand how to scale up the decentralized diagnosis and care for CL in Ethiopia and elsewhere.

## Supporting information

S1 File Checklists for Cryotherapy. (DOCX)

S2 File Checklists for skin slit smear. (DOCX)

S3 File PCR procedures. (DOCX)

S4 File Checklist. (DOCX)
