# Supplementary material for: Diagnosis of localized cutaneous leishmaniasis and treatment outcomes following carbon dioxide cryotherapy performed by health centre staff: A pilot implementation study in Ethiopia
Source: PLOS Glob Public Health. 2026 Sep 9;6(9):e0006930. doi: 10.1371/journal.pgph.0006930 (PMC13557335; doi:10.1371/journal.pgph.0006930)
Supplement: Supplementary file 1 [file pgph.0006930.s001.docx]

| **Effectiveness, Acceptability, and Feasibility of Decentralizing the Diagnosis and Carbon Dioxide-based Cryotherapy for the Treatment of Localized cutaneous leishmaniasis in the Health Centres of South Wollo Zone, Northeast Ethiopia** | | |  |
| --- | --- | --- | --- |
| **Skin slit smear observational checklist** | **Done** | **Not Done** |  |
| 1. **Introduction / Explanation of procedure** | | |  |
| 1.1. Welcomes/Greets individual |  |  |  |
| 1.2. Introduces self |  |  |  |
| 1.3. Explains the process for the laboratory procedure |  |  |  |
| 1.4. Seeks consent to proceed |  |  |  |
| 1.5. Asks individual if he/she has any questions |  |  |  |
| 1.6. Guides the individual to position comfortably |  |  |  |
| 2. **Preparation of procedure** | | |  |
| 2.1. Wear gloves |  |  |  |
| 2.2. Site selection for taking sample |  |  |  |
| 2.3. Cleans the lesion and surrounding skin with 70% alcohol |  |  |  |
| 2.4. Dries the lesion and surrounding skin with gauze |  |  |  |
| 2.5. Prepare the necessary materials |  |  |  |
| 2.6. Labelling slides (#02 slides the same labelling) |  |  |  |
| 3. **Sample taking procedures** | | |  |
| 3.1. Taking a skin slit from the lesion, **NOT** blood |  |  |  |
| 3.2. Allows skin slit site to get arrest bleeding (*Give rest between sample taking if the lesion is multiple*) |  |  |  |
| 3.3. Smearing technique (spreading the skin slit on the labelled slides) |  |  |  |
| 3.4. Samples (slides) air dry |  |  |  |
| 3.5. Staining technique with respective allowed time (*each staining steps*) |  |  |  |
| 3.6. Let slide dry |  |  |  |
| 4. **Parasite identification and reporting** |  |  |  |
| 4.1. Applies enough amount of emersion oil to the slides |  |  |  |
| 4.2. Start looking at the slides with using 10x |  |  |  |
| 4.3. Convers the objective lens to 100x |  |  |  |
| 4.4. Uses zigzag methods to look the slides |  |  |  |
| 4.5. Spend enough time to look the slides |  |  |  |
| 4.6 Result: *Positive  *Negative  *Other (Inconclusive result, invalid result) | | |  |
| 4.7. Appropriate reporting to the clinician | | |  |
| 4.8. Slides stored in the rack (*for microscopy and PCR*)   a) Positive b) Negative c) For PCR | | | |

**Observation checklist for skin slit smear**

**Points:**

1. Introduction/Explanation of procedure (Maximum 6)__________
2. Preparation of procedure (Maximum 6):___________
3. Sample taking procedures (Maximum 6): ___________
4. Parasite identification and reporting (Maximum 8):___________

**Total points (max. 26):__________**

**Total time spent for the procedure: _________________________**

(from greeting the patient to patient walking out)
