# Supplementary material for: Diagnosis of localized cutaneous leishmaniasis and treatment outcomes following carbon dioxide cryotherapy performed by health centre staff: A pilot implementation study in Ethiopia
Source: PLOS Glob Public Health. 2026 Sep 9;6(9):e0006930. doi: 10.1371/journal.pgph.0006930 (PMC13557335; doi:10.1371/journal.pgph.0006930)
Supplement: Supplementary file 2 [file pgph.0006930.s002.docx]

| **Cryotherapy observational checklist** | **Done** | **Not Done** |
| --- | --- | --- |
| 1. **Introduction / Explanation of procedure** | | |
| 1.1. Welcomes/Greets individual |  |  |
| 1.2. Introduces self |  |  |
| 1.3. Explains the process for cryotherapy |  |  |
| 1.4. Seeks consent to proceed |  |  |
| 1.5. Asks individual if he/she has any questions |  |  |
| 1.6. Guides the individual to position comfortably (if required) |  |  |
| 2. **Preparation of procedure** | | |
| 2.1. Cleans the lesion and surrounding skin |  |  |
| 2.2. Dries the lesion and surrounding skin with gauze |  |  |
| 2.3. Prepare the necessary materials |  |  |
| 2.4. Measure the size of the lesion |  |  |
| 2.5. Select the appropriate tips |  |  |
| 2.6. Adjust the timer |  |  |
| 3. **Application of treatment** | | |
| 3.1. Cool the tip of the cryo tip for 15seconds |  |  |
| 3.2. Apply cryotherapy for 40seconds: 2 mm outside the lesion margin (**A1**) |  |  |
| 3.3. Allows a freeze thaw for 20 seconds |  |  |
| 3.4. Repeats the freeze for 40seconds (**A2**) |  |  |
| 3.5. Repeat the procedures (3.1-3.4) on another section of the lesion(if the size of the lesion is >3cm) |  |  |
| 3.6. Wound dressing (if necessary) |  |  |
| 3.7. Explain the individual how to care and clean the wound |  |  |
| 3.8. Inform the individual about possible adverse effects |  |  |
| 4. **Further explanation/discharge/scheduling of next visit** |  |  |
| 4.1. Inform the individual about time for next cryotherapy session |  |  |
| 4.2 Schedules the individual for next visit |  |  |
| 4.3. Asks individual if she/he has any questions |  |  |
| 5. **During the follow up visit (**Treatment-experienced**):**  - Ask the adverse effects   - Pain   - Blistering   - Infection   - Color change | **Yes:** | **No:** |
|  | **[ ]**  **[ ]**  **[ ]**  **[ ]**  **[ ]** | **[ ]**  **[ ]**  **[ ]**  **[ ]**  **[ ]** |

**Observation of cryotherapy**

**Points:**

1. Introduction/Explanation of procedure (Maximum 6)__________
2. Preparation of procedure (Maximum 6):___________
3. Application of treatment (Maximum 8): ___________
4. Further explanation/discharge/scheduling of next visit (Maximum 3):___________
5. Treatment-experienced (Maximum 5)______________

**Total points (max. 23 (28 for treatment-experienced)):__________**

**Total time: _________________________**(from greeting the patient to patient walking out)

**Total time spent for the procedure: ______________________________
Number of freezes:____________________________________________**

**Duration of 1^st^ freeze: _________________________________________**

**Duration of 2^nd^ freeze: _________________________________________**

**Duration of 3^rd^ freeze: _________________________________________**

**Duration of 4^th^ freeze: _________________________________________**
