# Supplementary material for: Diagnosis of localized cutaneous leishmaniasis and treatment outcomes following carbon dioxide cryotherapy performed by health centre staff: A pilot implementation study in Ethiopia
Source: PLOS Glob Public Health. 2026 Sep 9;6(9):e0006930. doi: 10.1371/journal.pgph.0006930 (PMC13557335; doi:10.1371/journal.pgph.0006930)
Supplement: Supplementary file 3 [file pgph.0006930.s003.docx]

# **Detailed PCR Analysis steps**

# 1. Sample Collection and Verification

Slit skin smear (SSS) samples were collected from patients suspected to have Cutaneous leishmaniasis. The slides were prepared and read microscopically at the Ketetiya Health Center Laboratory. Laboratory technicians first read the slides, and confirmation was carried out at the field by Armauer Hansen Research Institute (AHRI) Laboratory Technicians. The confirmed slides were then stored for further molecular analysis.

# 2. Eluting Samples off Slides

To Prepare the samples for further molecular analysis, they were eluted and moved from the microscope slides into 1.5 mL Eppendorf tubes using the following process:

1. Slides were inverted onto absorbent paper to drain off any remaining immersion oil before processing.
2. 100 µL of ATL buffer (from the QIAGEN Blood and Tissue Kit) was placed over the sample area, and the slide was left undisturbed for 5 minutes to allow the ATL buffer to digest material from the slide surface.
3. The sample was then scratched using new pipet tips and transferred into a Pre-labelled 1.5 mL Eppendorf tube
4. 100 µL of PBS buffer was added to the slide for further Washing to collect remaining material transferred into a Pre-labelled 1.5 mL Eppendorf tube
5. Another 100 µL of PBS buffer was added to the slide to maximize sample recovery.
6. The final PBS wash was also transferred into the same 1.5 mL Eppendorf tube for a total sample volume of approximately 300 µL.

# 3. DNA Extraction

DNA was extracted with the QIAGEN Blood and Tissue Kit following exactly the manufacturer's instructions but with some adjustments during optimization.

1. 80 μL of Buffer ATL was added to the sample, followed by 20 μL of proteinase K. The mixture was vortexed thoroughly.
2. Samples were incubated overnight at 56°C with 500 RPM shaking for thorough tissue lysis.
3. After incubation, 200 μL of Buffer AL was added, and the mixture was vortexed thoroughly. The samples were incubated at 56°C for 10 more minutes.
4. 200 μL of 96-100% ethanol was added and vortexed. The sample was pipetted into a DNeasy Mini spin column housed in a 2 mL collection tube.
5. Centrifugation at 6,000 × g (8,000 rpm) for 1 minute was performed, and the flow-through was discarded.
6. The spin column was transferred to a fresh 2 mL collection tube, and 500 μL of Buffer AW1 was added. The column was centrifuged at 6,000 × g for 1 minute, and the flow-through discarded.
7. AW2 Buffer of 500 μL was added, and the column centrifuged for 3 minutes at 20,000 × g (14,000 rpm). Discarded the flow-through and placed the spin column into a new microcentrifuge tube.
8. 100 μL of AE was added directly in the center of the column membrane, incubated for 1 minute at room temperature, and then centrifuged at 6,000 × g for 1 minute.
9. The DNA eluate was collected and preserved at -20°C until additional molecular analysis.

# 4. PCR Amplification

For the molecular Detection of Leishmania species, PCR amplification of the Internal Transcribed Spacer (ITS) region 1 was performed. The PCR reaction was set as follows:

1. A master mix for a 50 µL reaction volume consisted of 25 µL of GoTaq® Green Master Mix, 1 µL of both primers (LISTR and L5.8S), and 18 µL of nuclease-free water.
2. 5 µL of DNA template was added to the reaction mixture, and the Volume was brought up to 50 µL. The PCR reaction was mixed and centrifuged briefly.
3. The PCR amplification conditions were:
   - Denaturation: 95°C for 15 minutes (initial cycle), 94°C for 1 minute (subsequent cycles).
   - Annealing: 53°C for 30 seconds.
   - Extension: 72°C for 1 minute.
   - Final extension: 72°C for 10 minutes.
   - A total of 35 cycles.
4. 5 µL of the PCR product were Run on 2% agarose gel in the presence of (2µL) SBR safe DNA gel stain. Stored the rest of the PCR product at 4℃.
5. The gel was examined under UV light to confirm the presence of the ITS-1 product.

# 6. Restriction Fragment Length Polymorphism (RFLP)

The positive PCR products were then characterized by restriction fragment length polymorphism (RFLP) to differentiate among Leishmania species. Reference *L.aethiopica L.tropica, L.donovani* and *L.infantum* species and Positive PCR product of the samples were incubated with the *Hha I* enzyme for restriction digest. HhaI enzyme does not have restriciton sites on *L.tropica, L.donovani and L.infantum* but cuts *L.aethiopica* at 171, 161 and 2 base pairs.

1. 0.5 µL of the HhaI restriction enzyme (20 U/µL) and 2 µL of the 10x reaction buffer was added to each 17 µL of PCR products and Reference strains. The reaction mixture was incubated for 4 hours at 37°C.
2. Agarose gels (2%) were made up with 2 µL of SYBR Safe DNA Gel Stain.
3. After incubation, 15 µL of the restriction digest was run on the gel, along with a molecular size marker(100 bp ladder) and reference strains.
4. The gel was Run at 80V for 90 minutes, and the fragments were visualized with UV light.
5. If the samples did not exhibit cleavage patterns consistent with those of the reference species L tropica, L.donovani, and L.infantum, they will be re-analyzed using the HaeIII enzyme for further investigation.

**Procedure_summary:**
The smear on the slide was blotted using absorbent paper to drain off any remaining immersion oil. Then, it was moistened by adding 100 µL of ATL buffer (from the QIAGEN Blood and Tissue Kit) for 5 minutes. A sterile pipet tip is used to scrub and transfer into a pre-labelled 1.5 mL Eppendorf tube, subsequently washed twice with 100 µL of phosphate-buffer saline (1X PBS, g/l of sodium chloride 8.0, potassium chloride 0.2, Di-sodium hydrogen phosphate 1.15, potassium di hydrogen phosphate 0.2 with ph7.3). The extraction was done using the QIAGEN Blood and Tissue Kit following the manufacturer’s instructions (QIAGEN GmbH, Hilden, LOT178029012, Germany). The ribosomal Internal Transcribed Spacer (ITS) region 1 was performed as described Schonian (17). In brief, a master mix for 50 µL consisted of 25 µL of GoTaq® Green Master Mix (Promega Corporation, Madison, WI 53711-5399 USA), 1 µL (from 10uM working concentration) of each primer (LISTR 5’-CTGGATCATTTTCCGATG-3’ and L5.8S 5’-TGATACCACTTATCGCACTT-3’), 5 µL of DNA (from a ~ 7.558 ng/ µL working) and 18 µL of nuclease-free water (Promega Corporation, Madison, WI 53711-5399 USA). The amplification conditions included, initial denaturation at 95°C for 15 minutes and 35 cycles of; denaturation at 94°C for 1 minute, annealing at 53°C for 30 seconds and extension at 72°C for 1 minute. A final extension of 10 minutes at 72°C. The 4 hours at 37°C water bath restriction enzyme digestion contained 0.5 µL of the *Hha*I restriction enzyme (20 U/µL), 2 µL of the 10x reaction buffer and 17 µL of PCR amplicons. The restriction fragments were subjected to electrophoresis in 2% agarose gels (Invitrogen, Life technologies, LOT 0001143663, USA) at 400mA, 80V for 90 minutes stained with SYBR Safe DNA gel stain (Invitrogen, Thermo Fisher scientific, USA) compared against 100bp molecular marker (Invitrogen,Thermo Fisher scientific, Balicts UAB, LOT3027016, Lithuania). The 240 and 88 bp band correspond to L. major, and the 162 bps correspond to *L. aethiopica*, while *L. tropica, L. infantum* and *L. donovani* does not have restriction sites for *HhaI* Enzyme
