# Supplementary material for: Diagnosis of localized cutaneous leishmaniasis and treatment outcomes following carbon dioxide cryotherapy performed by health centre staff: A pilot implementation study in Ethiopia
Source: PLOS Glob Public Health. 2026 Sep 9;6(9):e0006930. doi: 10.1371/journal.pgph.0006930 (PMC13557335; doi:10.1371/journal.pgph.0006930)
Supplement: Supplementary file 4 [file pgph.0006930.s004.docx]

Inclusivity in global research

**Ethical considerations, permits and authorship**

*This section is applicable to all research types.*

Provide details as to who granted permissions and/or consent for the study to take place in the Methods section of your manuscript. This should include the names of **all** ethics boards, governmental organizations, community leaders or other bodies that provided approval for the study. If individuals provided approval refer to these people by their role or title but do not list their name(s).

Ethical approval for the study was obtained from the Institutional Review Board (IRB) of the University of Gondar. Additional administrative permission was granted by the Armauer Hansen Research Institute (AHRI), which authorized data collection and study implementation within the study setting. All approvals were obtained prior to the commencement of the study. Where applicable, further permission to access specific departments or clinical units was obtained from the heads of the respective departments. All approvals were granted in their official capacity, and no personal names are disclosed in accordance with ethical reporting standards. The study was conducted in accordance with the approved protocol and relevant national and international ethical guidelines.

Reported on page number: 14

If there were any deviations from the study protocol after approval was obtained please provide details of these changes in the Methods section of your manuscript.

No deviations from the approved study protocol occurred after ethical approval was obtained. The study was conducted in full accordance with the approved protocol, and all procedures were implemented as originally specified.

Did this study involve local collaborators that are residents of the country where the research was conducted or members of the community studied? If you do not have any authors from said communities, please provide an explanation for this below.

Everyone listed as an author should meet PLOS’ criteria for authorship and all individuals who meet these criteria should be included in the author byline, rather than the acknowledgements. For further information please see the journal’s Authorship Policy.

All individuals listed as authors meet the authorship criteria defined by PLOS. Each author made substantial contributions to the conception or design of the study, data acquisition, analysis, contributed to drafting or critically revising the manuscript, approved the final version for submission, and agrees to be accountable for all aspects of the work. No individuals who meet these criteria have been omitted from the author byline.

Yes. The study involved local collaborators who are residents of the country where the research was conducted. These included clinicians, nurses, researchers and public health professionals affiliated with the participating institution, who contributed to study design, data collection, supervision of field activities, and interpretation of findings. Their involvement ensured that the research was contextually appropriate and aligned with local clinical and public health priorities. All collaborators are included in the authorship or acknowledged according to their level of contribution.

**Human subjects research (e.g. health research, medical research, cross-cultural psychology)**

Did you obtain written informed consent from a representative of the local community or region before the research took place? How did you establish who speaks for the community? Details of written informed consent obtained from study participants should be reported separately in the Methods section of your manuscript.

Since our research involved individual participants,thus written informed consent was obtained directly from each participant (or their legally authorized representative, where applicable) before enrollment. However, a written informed consent from a representative of the local community or region was not obtained because the study did not require community-level consent. Rather support letter was given from the regional public health institute. The study was conducted with approval from the relevant Institutional Review Board/Ethics Committee of the University of Gondar, and details of the participant informed consent process are described in the Methods section of the manuscript.

How did members of the local community provide input on the aims of the research investigation, its methodology, and its anticipated outcome(s)?

Members of the local community did not directly contribute to the development of the research aims, methodology, or anticipated outcomes. The study objectives and methodology were developed by the research team based on existing scientific evidence, the relevant literature, and local clinical and public health priorities. Although community members were not involved in the study design, all participants provided written informed consent prior to enrollment, and the research was conducted in accordance with approved ethical standards.

When engaging with the local community, how did you ensure that the informed consent documents and other materials could be understood by local stakeholders?

The informed consent documents and participant information sheets were prepared in English language that was clear and appropriate for the local population and were translated into the local language (Amharic). Trained study staff explained the study objectives, procedures, potential risks and benefits, confidentiality measures, and participants' rights in the participants' vernacular language. Participants were given the opportunity to ask questions, and informed consent was obtained only after the study team confirmed that they understood the information provided.

Will the findings of the research be made available in an understandable format to stakeholders in the community where the study was conducted (e.g. via a presentation, summary report, copies of publications, etc.)? Please provide details of how this will be achieved.

Yes. The findings of the study was shared with the participating health facilities clinical and laboratory staffs, health centre heads, district health office director, district and Zonal NTD officer, Zonal and regional NTD officers and relevant local health authorities through summary reports and presentations. In addition, the results were disseminated through scientific publications (***Global PLOS public Health***) and conference presentations. A plain-language summary of the key findings were made and available within the manuscript and other stakeholders to ensure that the results are communicated in an understandable and accessible format via paper based and the presentation was streemed using the local radio FM station . No information that could identify individual participants will be disclosed, yet.

**Non-human subjects research using specimens/ animals collected as part of the study, or those housed in archival collections. Examples include archaeology, paleontology, botany and zoology.**

Did the permission you obtained from a local authority to perform the study include an agreement on access to outputs and benefit sharing? This may include procedures to enable fair distribution of the benefits and resources arising from the research performed. Please include any details of Prior Informed Consent and Benefit Sharing Agreements obtained. These may be required by field-specific regulations, for example the Convention on Biological Diversity (CBD) and the associated Nagoya Protocol.

Yes. Permission to conduct the study was obtained from the relevant institutional and local health authorities before the research begun. The approval authorized the conduct of the study and the dissemination of its findings through scientific publications and presentations. Although no separate formal benefit-sharing agreement was established, the participating institutions will receive a summary of the study findings to inform clinical practice and future research.

If the material used in your study was imported, please A) provide the year it was imported and B) indicate whether permits were obtained to import/export the materials used, C) provide details of any permits obtained. If this information is not available, please indicate this.

No materials used in this study were imported or exported. Therefore, import or export permits were not required, and no such permits were obtained.

If you used archival specimens, please state how the material used in your study was acquired by the institute it is held in and provide details of any permits obtained for the original excavations/ sample collection. If this information is not available, please indicate this.

No archival specimens were used in this study. Therefore, information regarding acquisition of such materials and permits for original collection or excavation is not applicable.

How was the potential cultural significance of the materials collected in your study to local communities considered in your research design? Were Indigenous peoples and/or local researchers and institutions involved with archaeological excavations / collection of specimens? If so, please provide a description of their involvement.

The research was conducted in a clinical/public health setting using standard medical data and/or biological samples collected for diagnostic or research purposes, in accordance with approved ethical guidelines. No Indigenous peoples or cultural heritage stakeholders were involved in specimen collection activities.

If your manuscript includes photographs of human remains please indicate whether authors obtained permission from descendants or affiliated cultural communities to do so.

The manuscript does not include photographs of human remains. Therefore, permission from descendants or affiliated cultural communities was not required.
