# Supplementary material for: Diagnosis of localized cutaneous leishmaniasis and treatment outcomes following carbon dioxide cryotherapy performed by health centre staff: A pilot implementation study in Ethiopia
Source: PLOS Glob Public Health. 2026 Sep 9;6(9):e0006930. doi: 10.1371/journal.pgph.0006930 (PMC13557335; doi:10.1371/journal.pgph.0006930)
Supplement: Supplementary file 6 [file pgph.0006930.s006.docx]

**Diagnosis of localized cutaneous leishmaniasis and treatment outcomes following carbon dioxide cryotherapy performed by health centre staff : a pilot implementation study in Ethiopia**

**Responses point-by-point:**

**Journal Requirements:**

1. If you are submitting a clinical trial, it must be registered in a publicly accessible registry approved by the WHO or ICMJE (see <https://www.who.int/clinical-trials-registry-platform/network/primary-registries> and <http://www.icmje.org/about-icmje/faqs/clinical-trials-registration/>)
a) Please record the registration number in your submission Abstract, and include both the registration number and direct URL to the trial registration in the Methods section of your manuscript.

- *Dear editors, the study was registered in the Pan African Clinical Trials Registry with trial number PACTR202605616785682.* [*https://pactr.samrc.ac.za/TrialDisplay.aspx?TrialID=41111*](https://pactr.samrc.ac.za/TrialDisplay.aspx?TrialID=41111)
- *Page 2, Line 44-46*

b) Please also provide the complete date range for participant recruitment and follow-up in the Methods section of your manuscript.
   - *Dear editors, the data collection and case recruitment were conducted by clinicians at KHC between June 24, 2024 and January 01, 2025.*
   - *Page 7, line 144-145*
c) If you have not yet registered your trial in an appropriate registry, we now require you to do so and will need confirmation of the trial registry number before we can pass your paper to the next stage of review. Please include in the Methods section of your paper your reasons for not registering this study before enrolment of participants started. Please confirm that all related trials are registered by stating: “The authors confirm that all ongoing and related trials for this drug/intervention are registered”.

- *Dear editors, this trial was registered in the Pan African Clinical Trials Registry (PACTR202605616785682) after data collection and analysis. Because the study used a pragmatic/implementation design, we initially believed registration was unnecessary. After submitting to PLOS Global Public Health, the journal required registration, so we registered the trial post‑data collection and added the registration number. The authors confirm that all ongoing and related trials for this intervention are registered.*
- *Page 7, Line 165-171*

2. Please include a complete copy of PLOS’ questionnaire on inclusivity in global research in your revised manuscript. Our policy for research in this area aims to improve transparency in the reporting of research performed outside of researchers’ own country or community. The policy applies to researchers who have travelled to a different country to conduct research, research with Indigenous populations or their lands, and research on cultural artefacts. The questionnaire can also be requested at the journal’s discretion for any other submissions, even if these conditions are not met. Please find more information on the policy and a link to download a blank copy of the questionnaire here: <https://journals.plos.org/globalpublichealth/s/best-practices-in-research-reporting>. Please upload a completed version of your questionnaire as Supporting Information when you resubmit your manuscript.

- ***Dear Editors,*** *I have uploaded the questionnaire as supporting information. Page 10, Line 233, 261*

3. Please amend your detailed Financial Disclosure statement. This is published with the article. It must therefore be completed in full sentences and contain the exact wording you wish to be published.
I. Please clarify all sources of financial support for your study. List the grants, grant numbers, and organizations that funded your study, including funding received from your institution. Please note that suppliers of material support, including research materials, should be recognized in the Acknowledgements section rather than in the Financial Disclosure.

- *This study was supported through the Skin Health Africa Research Program, which is funded by the Research and Innovation for Global Health Transformation (RIGHT) Program of the National Institute for Health and Care Research (NIHR) (Grant Reference Number: NIHR200125). The funder had no role in the study design, data collection and analysis, decision to publish, or preparation of the manuscript.*
- *Page 21, Line 450-455*

II. State the initials, alongside each funding source, of each author to receive each grant. For example: "This work was supported by the National Institutes of Health (####### to AM; ###### to CJ) and the National Science Foundation (###### to AM)."

- ***K****indly refer to the financial statement presented in* ***Section 3.I*** *on* ***page 21, lines 450–455.***

III. State what role the funders took in the study. If the funders had no role in your study, please state: “The funders had no role in study design, data collection and analysis, decision to publish, or preparation of the manuscript.”

- ***K****indly refer to the financial statement presented in* ***Section 3.I*** *on* ***page 21, lines 450–455.***

If any authors received a salary from any of your funders, please state which authors and which funders.

- *Kindly refer to the financial statement presented in* ***Section 3.I*** *on* ***page 21, lines 450–455.***

4. In the online submission form, you indicated that “The data will be available upon request”.

- All PLOS journals now require all data underlying the findings described in their manuscript to be freely available to other researchers, either 
  1. In a public repository, 
  2. Within the manuscript itself, or 
  3. Uploaded as supplementary information.
- This policy applies to all data except where public deposition would breach compliance with the protocol approved by your research ethics board. If your data cannot be made publicly available for ethical or legal reasons (e.g., public availability would compromise patient privacy), please explain your reasons by return email and your exemption request will be escalated to the editor for approval. Your exemption request will be handled independently and will not hold up the peer review process, but will need to be resolved should your manuscript be accepted for publication. One of the Editorial team will then be in touch if there are any issues.
  - *The data supporting the findings of this study are available within the manuscript and its Supporting Information files. Due to privacy and confidentiality considerations, participant lesion photographs have been deposited in the institutional repository and are not publicly available. Access to these photographs granted by the Armauer Hansen Research Institution upon reasonable request and subject to applicable ethical and data protection requirements.*

5. We note that you have included your Figures within the body of your manuscript. Please remove the Figures from the body of your manuscript and upload them as separate Figure files in .tif or .eps format.

For more information about figure files please see our guidelines:

<https://journals.plos.org/globalpublichealth/s/figures> 
<https://journals.plos.org/globalpublichealth/s/figures#loc-file-requirements>

- *The figures (#2) are changed to tiff and uploaded to the submission*

6. Please ensure that your Ethics Statement is available in its entirety at the beginning of your Methods section, under a subheading 'Ethics Statement'.

- *Yes, sure the ethics statement is available under the subheading of 'Ethics Statement'.*
- *Page 13, Line 299-306*

7. We have noticed that you have a list of Supporting Information legends in your manuscript. However, there are no corresponding files uploaded to the submission. Please upload them as separate files with the item type 'Supporting Information'.
   - *Thank you very much for your valuable comments. We appreciate your suggestions. We confirm that all the supporting information has been uploaded along with the revised manuscript.*
   - *Page 26, Line 578-584*

- If the reviewer comments include a recommendation to cite specific previously published works, please review and evaluate these publications to determine whether they are relevant and should be cited. There is no requirement to cite these works unless the editor has indicated otherwise.
- Please review your reference list to ensure that it is complete and correct. If you have cited papers that have been retracted, please include the rationale for doing so in the manuscript text, or remove these references and replace them with relevant current references. Any changes to the reference list should be mentioned in the rebuttal letter that accompanies your revised manuscript. If you need to cite a retracted article, indicate the article’s retracted status in the References list and also include a citation and full reference for the retraction notice.
  - *Now we did not use the reference for these supplementary files used in the manuscript.*
  - *Up to my knowledge, we did not used the retracted paper(s)*

**Additional Editor Comments (if provided):**
Reviewers' comments:
Reviewer's Responses to Questions

- **Comments to the Author**

  1. Does this manuscript meet PLOS Global Public Health’s [publication criteria](https://track.editorialmanager.com/CL0/https:%2F%2Fjournals.plos.org%2Fglobalpublichealth%2Fs%2Fcriteria-for-publication/1/010f019efcbaca42-6e447f73-d285-49d3-8adb-085a2117223e-000000/u76pkVfey3PGG8eU-mVdOzIugd6czGnyACnBS4sSa7I=258)? Is the manuscript technically sound, and do the data support the conclusions? The manuscript must describe methodologically and ethically rigorous research with conclusions that are appropriately drawn based on the data presented.

Reviewer #1: Yes

Reviewer #2: Yes

- *Dear reviewer and editors, thank you for your review and comments. Yes, per the journal criteria and the comments (above) from the editors, I tried to address the areas to be improved in the methodology section including Ethics statement, financial disclosure and others.*
- *Page 14, Line 309-315*

2. Has the statistical analysis been performed appropriately and rigorously?

Reviewer #1: Yes

Reviewer #2: No

- *Thank you again for your valuable comments and constructive feedback. In the current manuscript, we have focused on reporting the effectiveness of the intervention as supported by the study data. However, we acknowledge that a comparative analysis with the current standard treatment recommended by the Federal Ministry of Health of Ethiopia would provide additional clinical context. Such an analysis is beyond the scope of the present manuscript but is planned for future work.*

3. Have the authors made all data underlying the findings in their manuscript fully available (please refer to the Data Availability Statement at the start of the manuscript PDF file)?

The [PLOS Data policy](https://track.editorialmanager.com/CL0/https:%2F%2Fjournals.plos.org%2Fglobalpublichealth%2Fs%2Fdata-availability/1/010f019efcbaca42-6e447f73-d285-49d3-8adb-085a2117223e-000000/uP8tk7NY71PCMa6G-0wALEKuAyB-txBzOC-Irrobna8=258) requires authors to make all data underlying the findings described in their manuscript fully available without restriction, with rare exception. The data should be provided as part of the manuscript or its supporting information, or deposited to a public repository. For example, in addition to summary statistics, the data points behind means, medians and variance measures should be available. If there are restrictions on publicly sharing data—e.g. participant privacy or use of data from a third party—those must be specified.

Reviewer #1: Yes

Reviewer #2: Yes

- *As discussed above (Editors sections- Question 4-), all the data underlying the findings which supporting the findings of this study are available within the manuscript and its Supporting Information files. Due to privacy and confidentiality considerations, participant lesion photographs have been deposited in the institutional repository and are not publicly available. Access to these photographs may be granted by the corresponding institution upon reasonable request and subject to applicable ethical and data protection requirements.*

4. Is the manuscript presented in an intelligible fashion and written in standard English?

PLOS Global Public Health does not copyedit accepted manuscripts, so the language in submitted articles must be clear, correct, and unambiguous. Any typographical or grammatical errors should be corrected at revision, so please note any specific errors here.

Reviewer #1: Yes

Reviewer #2: Yes

- *Thank you again for your comments and constructive feedback. In response to the reviewers' and editors' suggestions, we have also revised the manuscript to ensure that the spacing and formatting between sentences and paragraphs are consistent and uniform throughout the document.*

5. Review Comments to the Author

Please use the space provided to explain your answers to the questions above. You may also include additional comments for the author, including concerns about dual publication, research ethics, or publication ethics. (Please upload your review as an attachment if it exceeds 20,000 characters).

**Reviewer #1:** I commend the authors on for their outstanding working on evaluating CO2 cryotherapy in a non-specialized health center in Ethiopia. This manuscript highlights the rigorous efforts put into every stage of the study including engagement with the community, well-thought-out protocols, data collection forms, processes, analyses and dissemination of findings. The article is well written, no major concerns and suitable for publication. I have very minor suggestions for revision:

Overall, please ensure proportions are reported wherever a subset of the population is discussed. I will try and highlight as much of this as possible below:

Abstract
1) Line 47 - include proportion of laboratory confirmed CL (123 (78%) of denominator 158).

- *Thank you, Reviewer, for your thoughtful comments and valuable feedback, which have helped improve the quality and scientific rigor of our manuscript. In response to your suggestion, we have added the requested proportion to the manuscript (Page 2, Line 49; Page 14, Line 320; Page 15, Line 321;Page 16, Line 334).*
- Methods
  1) Lines 222-223 - include if there is, a reference for the where the established inclusion criteria have come from (ex. previously reported study reference 14)
  2)Lines 230-240 - similarly, include if any, a reference for where the established CO2 protocol may have come from (ex. previously reported study reference 14)
  *Dear reviewer,*
- *Thank you for your supportive and valuable feedback. We have added the requested references to the manuscript (Page 11, Line 232; Page 11, Line 247).*

Results
1)Lines 321-323 - please include reasons for ineligibility and refer to Figure 1

- *Dear reviewer,*
- *Thank you for your valuable feedback. Just I have added the reasons in the manuscript (Page 15-16, Line 335-341).*

Discussion
1)Line 375 - remove repeated "%"

- *Dear reviewer,*
- *Thank you for your careful and insightful observation. In response to your comment, we have removed the indicated text from the revised manuscript (Page 18, Lines 392).*

2)Lines 395-406 - please ensure line spacing is consistent

- *Apologies, I have corrected your comment (Page 19, Lines 412-424).*

**Reviewer #2:** This was a single-arm pilot study. The investigators did not calculate a formal sample size. The primary outcome was cure at Day 90 following the first application of CO2 cryotherapy. Descriptive statistics were used to summarize the demographic and clinical data of the study participants in the CO₂ intervention. They report the proportion of participants who were cured by Day 90 alongside changes in DLQI scores from baseline to Day 90.
The supplemental protocol is well written especially with the overall sample size needed for the 90% response and various levels of subjects needed at various precision considerations for detection of signs and symptoms
The paper is well written from a descriptively statistical perspective. Results follow from the analyses as seen in Figure 2 in that The dermatology expert assessed 68 of 77 (88.3%, 95% CI: 78.5-94.1%) as healed.
1. Please clarify the underlying distributional assumption of the confidence interval as it is not symmetrical.

The authors conclude that decentralized diagnosis and management with CO₂ cryotherapy can be successfully implemented in primary healthcare settings. The assumption being that one dermatology expert was the final quality control.

- *Thank you for this important observation and important comment. We have clarified this in the revised manuscript (Page 13, Lines 290-294).*
- 2. Was any consideration given to a random subset check of that final reading
- *Dear reviewer,*
- *Yes, even though a post hoc audit of a randomly selected subset of the final dataset was not performed, rigorous pre-analysis quality assurance procedures were implemented to ensure data integrity. These included standardized data collection protocols, internal consistency checks, and supervised validation of data entry. We have incorporated this clarification into the revised manuscript (Page 12, Lines 271–274).*

8. PLOS authors have the option to publish the peer review history of their article ([what does this mean?](https://track.editorialmanager.com/CL0/https:%2F%2Fjournals.plos.org%2Fglobalpublichealth%2Fs%2Feditorial-and-peer-review-process%23loc-peer-review-history/1/010f019efcbaca42-6e447f73-d285-49d3-8adb-085a2117223e-000000/9P6oPeMF6ikznLDOaKUG1aFATREvyJUGyTeQGWzCLik=258)). If published, this will include your full peer review and any attached files.

- *Thank you for your message. After careful consideration, I respectfully decline to publish the complete peer review history, including the correspondence and attached files exchanged between reviewers, editors, and authors. In my view, making these materials publicly available would not enhance the scientific integrity or quality of the manuscript. Therefore, I prefer that the peer review correspondence remains confidential.*
