# Supplementary material for: Diagnosis of localized cutaneous leishmaniasis and treatment outcomes following carbon dioxide cryotherapy performed by health centre staff: A pilot implementation study in Ethiopia
Source: PLOS Glob Public Health. 2026 Sep 9;6(9):e0006930. doi: 10.1371/journal.pgph.0006930 (PMC13557335; doi:10.1371/journal.pgph.0006930)
Supplement: Supplementary file 7 [file pgph.0006930.s005.docx]

***Comments response by response***

1) The in-house editorial staff feels that your study meets the World Health Organization definition of a clinical trial because it is a prospective study in which participants were assigned to cryotherapy to investigate the effects on cutaneous leishmaniasis treatment. Please change your manuscript’s article type to ‘Clinical Trial’ when you resubmit your manuscript."

- *Thank you dear*

*I did as suggested, you can check it on the submission*

2) Please upload a copy of the original ethics approval document you received from the London School of Hygiene & Tropical Medicine (29017) and the AHRI/ALERT Ethics Review Committee (Ref: PO/23/21) ethics committee. Where ethics approval was obtained from more than one study location, please provide approval document(s) from all of the sites. If the original document is in another language, please also provide an English translation.

- *Apologies for the error numbering*

*We were put the first ethical approval reference numbers before amendment. Now we put the current/updated version of the reference numbers of the respective ethical letters.*

3) Please report in the Methods section the day, month and year of the start and end of the recruitment period for this study in DD/MM/YYYY format.

- *Dear the Months and years were there in the setting subsection of the methods. Now as well we added the date we started. You can check it on the manuscript page 7;line 142-143.*

4) The ethical approval number(s) listed in the manuscript and/or submission metadata does not match the approval number on the ethical approval document you provided. Please ensure that all approval numbers are correct.

- *Yes, all the ethical approval letters and reference numbers are correct.*

5) Please provide the most recently approved Study Protocol, including any amendments with the date of the change noted, uploaded as a 'Supporting Information' file. If the original protocol is not in English, please also provide an English translation.

- *I submitted my PhD dissertation protocol, along with all required supporting documents, totaling 134 pages. The manuscript we are submitting is the third paper derived from this approved protocol. Therefore, I kindly request that it be considered within this context.*
- *In addition, University of Gondar is institution where I am attending my PhD journey, AHRI also a country research institute where supports the funding, and other resources and the LSHTM are the study funding institute.*
