# Supplementary material for: Diagnosis of localized cutaneous leishmaniasis and treatment outcomes following carbon dioxide cryotherapy performed by health centre staff: A pilot implementation study in Ethiopia
Source: PLOS Glob Public Health. 2026 Sep 9;6(9):e0006930. doi: 10.1371/journal.pgph.0006930 (PMC13557335; doi:10.1371/journal.pgph.0006930)
Supplement: Peer review history

# Author comment: Author response to Decision Letter 0

**DOI:** 10.1371/journal.pgph.0006930.r001

1 Jun 2026

Attachment Submitted filename: Point by point response.docx

# Peer review: Decision Letter 0

**DOI:** 10.1371/journal.pgph.0006930.r002

**Authors:** Andrés Henao-Martínez

24 Jun 2026

PGPH-D-26-01886

Diagnosis of localized cutaneous leishmaniasis and treatment outcomes following carbon dioxide cryotherapy performed by health centre staff: a pilot implementation study in Ethiopia

PLOS Global Public Health

Dear Dr. Tilahun Zewdu,

Thank you for submitting your manuscript to PLOS Global Public Health. After careful consideration, we feel that it has merit but does not fully meet PLOS Global Public Health’s publication criteria as it currently stands. Therefore, we invite you to submit a revised version of the manuscript that addresses the points raised during the review process.

Please submit your revised manuscript by Jul 24 2026 11:59PM. If you will need more time than this to complete your revisions, please reply to this message or contact the journal office at globalpubhealth@plos.org. When you're ready to submit your revision, log on to https://www.editorialmanager.com/pgph/ and select the 'Submissions Needing Revision' folder to locate your manuscript file.

Please include the following items when submitting your revised manuscript:

- A letter that responds to each point raised by the editor and reviewer(s). You should upload this letter as a separate file labeled 'Response to Reviewers'.
- A marked-up copy of your manuscript that highlights changes made to the original version. You should upload this as a separate file labeled 'Revised Manuscript with Track Changes'.
- An unmarked version of your revised paper without tracked changes. You should upload this as a separate file labeled 'Manuscript'.

Guidelines for resubmitting your figure files are available below the reviewer comments at the end of this letter.

As the corresponding author, your ORCID iD is verified in the submission system and will appear in the published article. PLOS supports the use of ORCID, and we encourage all coauthors to register for an ORCID iD and use it as well. Please encourage your coauthors to verify their ORCID iD within the submission system before final acceptance, as unverified ORCID iDs will not appear in the published article. *Only*  the individual author can complete the verification step; PLOS staff *cannot*  verify ORCID iDs on behalf of authors.

We look forward to receiving your revised manuscript.

Kind regards,

Andrés F. Henao-Martínez, M.D.

Academic Editor

PLOS Global Public Health

**Journal Requirements:**

1. If you are submitting a clinical trial, it must be registered in a publicly accessible registry approved by the WHO or ICMJE (see https://www.who.int/clinical-trials-registry-platform/network/primary-registries and http://www.icmje.org/about-icmje/faqs/clinical-trials-registration/)

a) Please record the registration number in your submission Abstract, and include both the registration number and direct URL to the trial registration in the Methods section of your manuscript.

b) Please also provide the complete date range for participant recruitment and follow-up in the Methods section of your manuscript.

c) If you have not yet registered your trial in an appropriate registry, we now require you to do so and will need confirmation of the trial registry number before we can pass your paper to the next stage of review. Please include in the Methods section of your paper your reasons for not registering this study before enrolment of participants started. Please confirm that all related trials are registered by stating: “The authors confirm that all ongoing and related trials for this drug/intervention are registered”.

2. Please include a complete copy of PLOS’ questionnaire on inclusivity in global research in your revised manuscript. Our policy for research in this area aims to improve transparency in the reporting of research performed outside of researchers’ own country or community. The policy applies to researchers who have travelled to a different country to conduct research, research with Indigenous populations or their lands, and research on cultural artefacts. The questionnaire can also be requested at the journal’s discretion for any other submissions, even if these conditions are not met. Please find more information on the policy and a link to download a blank copy of the questionnaire here: https://journals.plos.org/globalpublichealth/s/best-practices-in-research-reporting. Please upload a completed version of your questionnaire as Supporting Information when you resubmit your manuscript.

3. Please amend your detailed Financial Disclosure statement. This is published with the article. It must therefore be completed in full sentences and contain the exact wording you wish to be published.

i. Please clarify all sources of financial support for your study. List the grants, grant numbers, and organizations that funded your study, including funding received from your institution. Please note that suppliers of material support, including research materials, should be recognized in the Acknowledgements section rather than in the Financial Disclosure.

ii. State the initials, alongside each funding source, of each author to receive each grant. For example: "This work was supported by the National Institutes of Health (####### to AM; ###### to CJ) and the National Science Foundation (###### to AM)."

iii. State what role the funders took in the study. If the funders had no role in your study, please state: “The funders had no role in study design, data collection and analysis, decision to publish, or preparation of the manuscript.”

iv. If any authors received a salary from any of your funders, please state which authors and which funders.

4. In the online submission form, you indicated that “The data will be available upon request”.

All PLOS journals now require all data underlying the findings described in their manuscript to be freely available to other researchers, either

1. In a public repository,

2. Within the manuscript itself, or

3. Uploaded as supplementary information.

This policy applies to all data except where public deposition would breach compliance with the protocol approved by your research ethics board. If your data cannot be made publicly available for ethical or legal reasons (e.g., public availability would compromise patient privacy), please explain your reasons by return email and your exemption request will be escalated to the editor for approval. Your exemption request will be handled independently and will not hold up the peer review process, but will need to be resolved should your manuscript be accepted for publication. One of the Editorial team will then be in touch if there are any issues.

5. We note that you have included your Figures within the body of your manuscript. Please remove the Figures from the body of your manuscript and upload them as separate Figure files in .tif or .eps format.

For more information about figure files please see our guidelines:

https://journals.plos.org/globalpublichealth/s/figures

https://journals.plos.org/globalpublichealth/s/figures#loc-file-requirements

6. Please ensure that your Ethics Statement is available in its entirety at the beginning of your Methods section, under a subheading 'Ethics Statement'.

7. We have noticed that you have a list of Supporting Information legends in your manuscript. However, there are no corresponding files uploaded to the submission. Please upload them as separate files with the item type 'Supporting Information'.

If the reviewer comments include a recommendation to cite specific previously published works, please review and evaluate these publications to determine whether they are relevant and should be cited. There is no requirement to cite these works unless the editor has indicated otherwise.

Please review your reference list to ensure that it is complete and correct. If you have cited papers that have been retracted, please include the rationale for doing so in the manuscript text, or remove these references and replace them with relevant current references. Any changes to the reference list should be mentioned in the rebuttal letter that accompanies your revised manuscript. If you need to cite a retracted article, indicate the article’s retracted status in the References list and also include a citation and full reference for the retraction notice.

**Additional Editor Comments (if provided):**

[Note: HTML markup is below. Please do not edit.]

Reviewers' comments:

Reviewer's Responses to Questions

**Comments to the Author**

1. Does this manuscript meet PLOS Global Public Health’s publication criteria?

Reviewer #1: Yes

Reviewer #2: Yes

**********

2. Has the statistical analysis been performed appropriately and rigorously?

Reviewer #1: Yes

Reviewer #2: No

**********

3. Have the authors made all data underlying the findings in their manuscript fully available (please refer to the Data Availability Statement at the start of the manuscript PDF file)?

The PLOS Data policy

Reviewer #1: Yes

Reviewer #2: Yes

**********

4. Is the manuscript presented in an intelligible fashion and written in standard English?

Reviewer #1: Yes

Reviewer #2: Yes

**********

**Reviewer #1:**  I commend the authors on for their outstanding working on evaluating CO2 cryotherapy in a non-specialized health center in Ethiopia. This manuscript highlights the rigorous efforts put into every stage of the study including engagement with the community, well-thought-out protocols, data collection forms, processes, analyses and dissemination of findings. The article is well written, no major concerns and suitable for publication. I have very minor suggestions for revision:

Overall, please ensure proportions are reported wherever a subset of the population is discussed. I will try and highlight as much of this as possible below:

Abstract

1)Line 47 - include proportion of laboratory confirmed CL (123 (78%) of denominator 158)

Methods

1) Lines 222-223 - include if there is, a reference for the where the established inclusion criteria have come from (ex. previously reported study reference 14)

2)Lines 230-240 - similarly, include if any, a reference for where the established CO2 protocol may have come from (ex. previously reported study reference 14)

Results

1)Lines 321-323 - please include reasons for ineligibility and refer to Figure 1

Discussion

1)Line 375 - remove repeated "%"

2)Lines 395-406 - please ensure line spacing is consistent

**Reviewer #2:**  This was a single-arm pilot study. The investigators did not calculate a formal sample size. The primary outcome was cure at Day 90 following the first application of CO2 cryotherapy. Descriptive statistics were used to summarize the demographic and clinical data of the study participants in the CO₂ intervention. They report the proportion of participants who were cured by Day 90 alongside changes in DLQI scores from baseline to Day 90.

The supplemental protocol is well written especially with the overall sample size needed for the 90% response and various levels of subjects needed at various precision considerations for detection of signs and symptoms

The paper is well written from a descriptively statistical perspective. Results follow from the analyses as seen in Figure 2 in that The dermatology expert assessed 68 of 77 (88.3%, 95% CI: 78.5-94.1%) as healed.

1. Please clarify the underlying distributional assumption of the confidence interval as it is not symmetrical.

The authors conclude that decentralized diagnosis and management with CO₂ cryotherapy can be successfully implemented in primary healthcare settings. The assumption being that one dermatology expert was the final quality control.

2. Was any consideration given to a random subset check of that final reading

**********

what does this mean?). If published, this will include your full peer review and any attached files.

**Do you want your identity to be public for this peer review?**  If you choose “no”, your identity will remain anonymous but your review may still be made public.

For information about this choice, including consent withdrawal, please see our Privacy Policy

Reviewer #1: No

Reviewer #2: No

**********

[NOTE: If reviewer comments were submitted as an attachment file, they will be attached to this email and accessible via the submission site. Please log into your account, locate the manuscript record, and check for the action link "View Attachments". If this link does not appear, there are no attachment files.]

**Figure Resubmissions:**

# Author comment: Author response to Decision Letter 1

**DOI:** 10.1371/journal.pgph.0006930.r003

1 Jul 2026

Attachment Submitted filename: Response to Reviewers_25.06.2026FTZ.docx

# Editor report: Decision Letter 1

**DOI:** 10.1371/journal.pgph.0006930.r004

**Authors:** Andrés Henao-Martínez

6 Jul 2026

Diagnosis of localized cutaneous leishmaniasis and treatment outcomes following carbon dioxide cryotherapy performed by health centre staff: a pilot implementation study in Ethiopia

PGPH-D-26-01886R1

Dear Mr Tilahun Zewdu,

We are pleased to inform you that your manuscript 'Diagnosis of localized cutaneous leishmaniasis and treatment outcomes following carbon dioxide cryotherapy performed by health centre staff: a pilot implementation study in Ethiopia' has been provisionally accepted for publication in PLOS Global Public Health.

Before your manuscript can be formally accepted you will need to complete some formatting changes, which you will receive in a follow up email. A member of our team will be in touch with a set of requests.

Please note that your manuscript will not be scheduled for publication until you have made the required changes, so a swift response is appreciated.

IMPORTANT: The editorial review process is now complete. PLOS will only permit corrections to spelling, formatting or significant scientific errors from this point onwards. Requests for major changes, or any which affect the scientific understanding of your work, will cause delays to the publication date of your manuscript.

If your institution or institutions have a press office, please notify them about your upcoming paper to help maximize its impact. If they'll be preparing press materials, please inform our press team as soon as possible -- no later than 48 hours after receiving the formal acceptance. Your manuscript will remain under strict press embargo until 2 pm Eastern Time on the date of publication. For more information, please contact globalpubhealth@plos.org.

Thank you again for supporting Open Access publishing; we are looking forward to publishing your work in PLOS Global Public Health.

Best regards,

Andrés F. Henao-Martínez, M.D.

Academic Editor

PLOS Global Public Health

***********************************************************

Reviewer Comments (if any, and for reference):
